# Identification of Genetic Associations and Functional Polymorphisms of *SAA1* Gene Affecting Milk Production Traits in Dairy Cattle

**DOI:** 10.1371/journal.pone.0162195

**Published:** 2016-09-09

**Authors:** Shaohua Yang, Yahui Gao, Shengli Zhang, Qin Zhang, Dongxiao Sun

**Affiliations:** College of Animal Science and Technology, Key Laboratory of Animal Genetics and Breeding of Ministry of Agriculture, National Engineering Laboratory of Animal Breeding, China Agricultural University, Beijing, 100193, China; Universite Paris-Sud, FRANCE

## Abstract

Our initial RNA sequencing (RNA-seq) revealed that the *Serum amyloid A1* (*SAA1*) gene was differentially expressed in the mammary glands of lactating Holstein cows with extremely high versus low phenotypic values of milk protein and fat percentage. To further validate the genetic effect and potential molecular mechanisms of *SAA1* gene involved in regulating milk production traits in dairy cattle, we herein performed a study through genotype-phenotype associations. Six identified SNPs were significantly associated with one or more milk production traits (0.00002< *P* < 0.0025), providing additional evidence for the potential role of *SAA1* variants in milk production traits in dairy cows. Subsequently, both luciferase assay and electrophoretic mobility shift assay (EMSA) clearly demonstrated that the allele A of g.-963C>A increased the promoter activity by binding the PARP factor while allele C did not. Bioinformatics analysis indicated that the secondary structure of *SAA* protein changed by the substitution A/G in the locus c. +2510A>G. Our findings were the first to reveal the significant associations of the *SAA1* gene with milk production traits, providing basis for further biological function validation, and two identified SNPs, g.-963C>A and c. +2510A>G, may be considered as genetic markers for breeding in dairy cattle.

## Introduction

The dairy industry provides milk and dairy products for the human diet and it plays a key role in agricultural economy [1–2]. As the most important economic traits, an improvement in milk production traits continues to be the most profitable breeding goal [3–4]. Although classical DNA-based marker technology has expedited the genetic improvement of animal performance [5], the implementation of new strategies for investigating genes underlying complex traits could facilitate the improvement in selection accuracies.

With the maturing of next generation sequencing (NGS) technologies, RNA sequencing (RNA-seq) has been rapidly recognized as an accurate transcriptome profiling system for gene discovery. Several studies have been reported to identify key genes for the bovine transcriptome using RNA-seq techniques [6–10]. Our recent RNA-seq research has identified 31 differentially expressed genes in the mammary glands between the high and low groups for milk protein and fat percentage of lactating Holstein cows [11]. Of these, *SAA1* was significantly down-regulated in the Holstein cows with higher milk protein and fat percentage [11]. However, whether the *SAA1* gene has large genetic effects on milk production traits in dairy cattle has been undetermined yet. Further, it was found that the *SAA1* is located only 24.8 Kb and 4.2 Mb, respectively, from the two SNPs (ARS-BFGL-NGS-24998 and UA-IFASA-8605) which were significantly associated with fat percentage and protein percentage [12]. In addition, *SAA1* is also adjacent to a previously reported QTL for milk production traits [13]. Although its biological functions are debated, the wide species distribution of highly homologous *SAA1* gene has been observed in mammals. Recently, several studies have demonstrated that *SAA* concentrations were associated with bovine mastitis [14–17]. As a mediator of lipid transportation, *SAA1* is largely associated with the high-density lipoproteins (HDLs) [18]. By integrating the aforementioned evidences [11–13], we anticipate that the *SAA1* gene could be a promising functional gene for milk production traits in dairy cattle.

The aims of this study were to further validate the genetic effect and potential molecular mechanisms of *SAA1* gene involved in regulating milk production traits in dairy cattle. Herein, we detected genetic polymorphisms of the *SAA1* and tested the relationship between these variants with milk production traits in an independent dairy cattle population. The identified SNP loci were considered as preliminary foundation for further functional analysis and eventually unraveling the causal mutations implicated in milk production ability in dairy cattle. Furthermore, dual-luciferase reporter assay and bioinformatics analysis identified two potentially functional SNPs that influence the promoter activity and the protein structure of *SAA1*, respectively. Our study firstly identified the genetic variants in *SAA1* gene may be important genetic factors for milk production traits in dairy cattle and will be available for marker-assisted breeding based on further validation.

## Materials and Methods

All protocols for collection of frozen semen and blood samples of experimental individuals and phenotypic observations were approved by the Institutional Animal Care and Use Committee (IACUC) at China Agricultural University (Permit Number: DK996).

### Animals

A total of 717 Chinese Holstein cows from 12 sire families were used in this study. Each sire had between 47 and 134 daughters with approximately 60 daughters on average per sire. These cows were raised in 16 dairy farms (Sanyuanlvhe Dairy Farming Center), Beijing, China, where standard performance testing for dairy herd improvement (DHI) has been regularly conducted since 1999. Estimated breeding values (EBVs) for five milk production traits, namely milk yield (MY), protein yield (PY), fat yield (FY), protein percentage (PP) and fat percentage (FP), were used as ‘‘phenotypes” in the association study. These EBVs were previously calculated based on a multiple trait random regression test-day model using the software RUNGE by the Dairy Data Center of Dairy Association of China (http://www.holstein.org.cn/).

### DNA Extraction

Genomic DNAs were extracted from whole blood samples of cows by DP318 Blood DNA Kit (Tiangen Biotech Co., China) according to the manufacturer’s instructions and from semen samples of the sires using the routine salt-out procedures [19]. The quantity and quality of extracted DNAs were measured by NANODROP 2000 Spectrophotometer (Thermo Scientific, DE, USA).

### SNP Identification and Genotyping

Based on the genomic sequence of the bovine *SAA1* (GenBank accession no.: AC_000186.1), eight pairs of primers were designed with Oligo 6.0 software to amplify the entire coding region and 1920 bp of 5’ flanking sequences (Table 1). A DNA pool (50 ng/mL) was constructed with equal DNA concentration for each of the 12 sires. PCR conditions were as follows: initial denaturation at 95°C for 5 min, followed by 36 cycles of 30s at 95°C, annealing at 60°C for 30s, extension at 72°C for 30s, and a final extension at 72°C for 5 min. The PCR products were directly sequenced using ABI3730xl DNA analyzer (Applied Biosystems, CA, USA), and the sequences were compared by DNAMAN 6.0 (Lynnon Biosoft, USA) and Chromas 2.0 (Technelysium, Helensvale, Australia) to search SNPs. The identified SNPs were further genotyped for all the individuals using the iPLEX MassARRAY system (Sequenom Inc., CA, USA).

**Table 1:** Primers and PCR condition applied for pooled DNA sequencing for the *SAA1* gene.

Primers	Location	Primers sequences (5’-3’)	Annealing temperature	PCR products
P1	promoter region	F: CCATTGCCTTCTCCGATTTA	60°C	339 bp
		R: TCAGGGCAACAAATCATCAG		
P2	promoter region	F: CTGATGATTTGTTGCCCTGA	58°C	372bp
		R: GTTGCCATTTCCTTCTCCAG		
P3	promoter region	F: CTGGAGAAGGAAATGGCAAC	59°C	422 bp
		R: TTGTCTGCTGCATTTTCCTG		
P4	promoter	F: CAGGAAAATGCAGCAGACAA	60°C	690 bp
		R: TTGTCCTCATCCACAGGTCA		
P5	exon1	F: CACACCTTCTGTCTGGCTCA	59°C	555 bp
		R: CATTTCCCTTGTGCCACTCT		
P6	exon2	F: ATCAAAGCCATCTGGGTGAC	60°C	513 bp
		R: ATGATGCGCGTTCTTTCTCT		
P7	exon3	F: GCTATGACCACAGGCTGAAA	59°C	387bp
		R: CAGGAGGGTGGGTAAGAGAA		
P8	exon4	F: ACCCCTTCTCTATGGGTGCT	60°C	589 bp
		R: TCCCAGAACCAAACACACAA		

### Haplotype Analysis

Sporadic missing genotype data were imputed using Beagle 3.2 program [20]. Linkage disequilibrium (LD) extent between all SNP pairs and haplotype blocks were estimated using the software Haploview 4.2 (Broad Institute of MIT and Harvard, Cambridge, MA, USA).

### Association Analyses

Association analyses were conducted using SAS 9.1.3 (SAS Institute Inc., Cary, NC, USA) between the seven SNP genotypes or haplotypes and the five lactation traits, based on the following single-trait linear regression model:

y=1μ+Xb+Za+e

For each trait, ***y*** is a vector of “phenotypes” (EBVs) for all the 717 daughters, μ is the overall mean, b is a vector of SNP genotype or haplotype effects (i.e., fixed-effects), *X* is the design vector (0 or 1) linking a SNP genotype or haplotype to its genetic effect, $\alpha ∼N(0,A\sigma_{\alpha }^{2})$ is vector of (random) additive genetic value of each animal (not including the effects of SNP genotypes or haplotypes) with $\sigma_{\alpha }^{2}$ being the additive genetic variance, Z is a diagonal incidence (0 or 1) matrix that links each animal additive genetic value to its “phenotype”, and $\text{e}∼\text{N}(0,\text{W}\sigma_{e}^{2})$ is vector of residual terms with $\sigma_{\text{e}}^{2}$ being the residual variance. In the above, *A* is an additive genetic relationship numerator matrix, which was computed by tracing the pedigree back to three generations of 1,619 involved individuals, and W is a diagonal matrix with each diagonal element being equal to the inverse of the reliability of EBV for the corresponding individual [21]. Variance components were estimated based on the data of 30,000 Chinese Holstein cows in Beijing area by using the DMU package version 6 (University of Aarhus, Foulum, Denmark).

Bonferroni correction was performed for multiple t-testing through dividing the significance level by the number of tests, which is the number of SNPs being investigated. Hence, an association was considered statistically significant from being null effect if a raw *P* value is less than 0.05/N, where N is the number of SNP loci.

### Recombinant Plasmid Construction

To directly detect the allele-specific effects of the SNPs g.-963C>A and g.-781A>G on the promoter activity, three luciferase reporter gene fragments corresponding to the *SAA1* promoter (-973 to +46, +1 represents transcription start site) were synthesized, representing three haplotypes constructed by g.-963C>A and g.-781A>G (CA; CG; AG). As designed, the three promoter fragments, including the KpnI and BglII restriction sites at the 5’ and 3’ termini, respectively, were synthesized (Genewiz, Suzhou, China) and cloned into the pGL4.14 Luciferase Assay Vector (Promega). The three plasmid constructs were sequenced to confirm the integrity of each construct’s insertions. All plasmids were purified with the Endo-free Plasmid Maxi Kit (ComWin Biotech, Beijing, China).

### Cell Culture and Luciferase Assay

Human Embryonic Kidney (HEK) -293T cells (provided by the Department of Biochemistry, Guangdong Medical, China) were maintained in Dulbecco’s modified Eagle’s medium (DMEM) containing 10% heat-inactivated fetal bovine serum (FBS), 100 mg streptomycin and 100 U/ml penicillin at 5% CO_2_ and 37°C. Cells were seeded in 24-well plates at approximately 2×10^5^ cells per /well before transfection. Cells were transiently transfected using Lipofectamine 2000 (Invitrogen, CA, USA) according to the manufacturer’s protocol. For each cell, 0.9 g of the construct was co-transfected along with 0.1 g of pRL-TK renilla luciferase reporter vector (Promega) to control transfection efficiency and variation in plating. All the experiments were performed in three replicates for each construct.

Approximately 48 h after transfection, cells were harvested and the activity of both firefly and Renilla luciferases were measured using a Dual-Luciferase Reporter Assay System (Promega) on a Modulus microplate multimode reader (Turner Biosystems, CA, USA). The normalized luciferase data (firefly/renilla) were used to perform the average statistics of three replicates.

### Electrophoretic Mobility-Shift Assay (EMSA)

To assess the alteration of the DNA-binding ability caused by the regulatory mutations, EMSA was performed using biotinylated oligonucleotide probes containing variant g.-963C>A (AGGATGAAGCACAAG**A/C**AACATTTTCTT). Nuclear extracts from the mammary gland tissue of lactating Holstein cows were prepared using a NE-PER^®^ Nuclear and Cytoplasmic Extraction Reagents (Thermo Scientific).

Fifteen μg of crude nuclear protein were incubated for 20 min at room temperature in a 15-μl binding reaction system, which included 1.5μl 10×binding buffer, 1.5 μl poly(dI-dC) (1.0ug/ul), and ddH_2_O to meet a final volume of 10 μl. For each reaction, 0.6 μl Bio-wild probe or Bio-mutant probe (500fM) was added, and the reaction was incubated for 20 min at room temperature. As negative controls for the competition binding, 100-fold excess of non-radio labelled probes were used as competitors before the labeled probe and incubated for 20 min. Finally, the protein-DNA complexes were separated by electrophoresis in a native 6% DNA retardation gel at 150 V for 4 hours.

### Bioinformatics Analysis

Firstly, putative differential transcription factor binding sites (TFBSs) of the genetic variants in promoter region were predicted with the online software TFSEARCH (http://mbs.cbrc.jp/research/db/TFSEARCH.html). Also, secondary structures of the bovine *SAA1* mRNA were predicted by the software RNA structure4.6. For the missense mutation c. +2510A>G, the potential impact on the structure alteration of the *SAA* protein was predicted by SOPMA SERVER (http://npsa-pbil.ibcp.fr/) while functional prediction was performed by the web server tool SIFT (http://sift.jcvi.org/www/SIFT.html).

## Results

### SNP Identification

**Fig 1:**
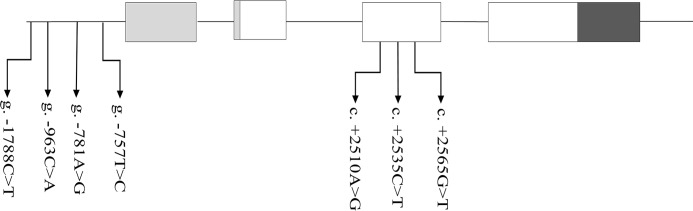
Positions of the seven identified SNPs in the *SAA1* gene. The gray, black and white bars represent 5’ UTR, 3’ UTR and exons, respectively; intervals represent introns.

A total of 7 SNPs for *SAA1* were discovered (Fig 1), including 4 novel SNPs in the promoter regions (g.-1788C>T, g.-963C>A, g.-781A>G and g.-757T>C) and 3 previously reported SNPs in coding region, c. +2510A>G (rs378661311), c.+2535C>T (rs383809871) and c.+2565G>T (rs208890658). Of these, c. +2510 A>G was predicted to result in an amino acid replacement of Gly with Asp. All the 7 SNPs were finally individually genotyped for the association analysis. Chi-square tests showed that all these SNPs were in Hardy-Weinberg equilibrium (*P* >0.05) in this experimental dairy population. The genotypic and allele frequencies were summarized in Table 2.

**Table 2:** Information about the seven identified SNPs of the *SAA1* gene and their genotypic and allelic frequencies.

SNPs	Location	Amino acid substitution	Position in UMD_3.1	GenBank no.	Genotype	Genotype frequencies	Allele	Allele frequencies
g.-1788C>T	Promoter		26694425 bp	ss831884868	CC	0.692	C	0.834
					TC	0.286	T	0.166
					TT	0.022		
g.-963C>A	Promoter		26694650 bp	ss831884869	CC	0.693	C	0.836
					CA	0.286	A	0.164
					AA	0.021		
g.-781 A>G	Promoter		2694832 bp	ss831884870	AA	0.375	A	0.630
					AG	0.509	G	0.370
					GG	0.116		
g.-757 T>C	Promoter		26694856 bp	ss831884871	TT	0.307	T	0.548
					CT	0.482	C	0.452
					CC	0.211		
c.+2510 A>G	exon3	D48G	26698026bp	rs133033048	AA	0.689	A	0.834
					AG	0.290	G	0.166
					GG	0.021		
c.+2535 C>T	exon3	R56R	26698052 bp	rs133033048	CC	0.404	C	0.632
					CT	0.456	T	0.368
					TT	0.140		
c.+2565 G>A	exon3	P66P	26698082 bp	rs467989313	GG	0.694	G	0.838
					GT	0.289	T	0.162
					TT	0.017		

### Association Analyses

Association analyses between EBVs of five milk production traits and the 7 SNPs are shown in Table 3. Of the 7 SNPs, after Bonferroni correction for multiple testing, 6 SNPs (g.-1788C>T, g.-963C>A, g.-781A>G, c. +2510A>G, c. +2535C>T and c. +2565G>T) remained significantly associated with MY (0.00006< *P* <0.0064) and PY (0.00002 < *P* < 0.0013), one SNP c. +2510A>G was strongly associated with FP (*P* = 0.0066). However, no significant associations were found with the SNP g.-757 T>C.

**Table 3:** Estimated association effects of 7 SNPs in the *SAA1* gene on milk production traits in Chinese Holsteins (LSM±SE).

SNPs	Genotype	MY^1^	FY	FP	PY	PP
g.-1788C>T	CC(496)	430.61±59.38^B^	4.77±2.68	-0.07±0.027	11.39±1.88^A^	0.003±0.005
	TC(205)	225.65±66.56^A^	5.63±2.92	-0.02±0.030	6.16±2.04^B^	0.001±0.007
	TT(16)	396.65±155.11^B^	4.99±6.26	-0.07±0.068	8.34±4.38^B^	-0.029±0.024
	P value	0.0019*	0.9066	0.0295	0.0006**	0.4104
g.-963C>A	AA(20)	231.16±155.58^A^	0.99±6.43	-0.05±0.070^a^^b^	3.35±4.49^A^	-0.027±0.025
	CA(196)	232.19±66.66^A^	3.26±2.92	-0.01±0.030^a^	6.38±2.04^A^	0.004±0.009
	CC(501)	399.08±59.37^B^	4.60±2.68	-0.07±0.027^b^	11.43±1.88^B^	0.006±0.008
	P value	0.0025^*^	0.5459	0.0139	0.0003**	0.6489
g.-781 A>G	AA(284)	470.38±64.67^A^	6.50±2.87	-0.08±0.029	13.70±2.01^A^	0.006±0.009
	AG(335)	297.89±60.74^A^	5.17±2.72	-0.04±0.027	8.24±1.91^B^	0.005±0.008
	GG(98)	180.54±83.64^B^	-0.11±3.55	-0.05±0.037	4.26±2.48^B^	-0.008±0.012
	P value	0.00006^**^	0.0779	0.1627	0.00007**	0.9657
g.-757 T>C	TT(211)	446.34±67.71	4.77±3.78	-0.10±0.031	8.44±2.19	-0.013±0.009
	CT(332)	305.19±61.82	4.98±2.76	-0.04±0.028	8.84±1.93	-0.008±0.008
	CC(145)	290.80±72.31	3.27±2.98	-0.05±0.033	12.34±2.09	0.001±0.010
	P value	0.0117	0.7137	0.0623	0.0193	0.4695
c.+2510 A>G	AA(499)	406.91±59.38^A^	4.67±2.68	-0.07±0.027^A^	11.64±1.88^A^	0.006±0.008
	AG(198)	216.70±66.46^B^	6.09±2.92	-0.01±0.030^B^	5.99±2.04^B^	0.003±0.009
	GG(20)	231.32±159.58^A^^B^	0.99±6.43	-0.05±0.070^A^^B^	3.37±4.49^B^	-0.027±0.025
	P value	0.0004**	0.6021	0.0066*	0.00002**	0.6148
c.+2535 C>T	CC(496)	393.24±59.40^A^	4.74±2.68	-0.07±0.027^b^	11.31±1.88^A^	0.005±0.008
	TC(205)	239.88±66.43^B^	5.89±2.92	-0.01±0.030^a^	6.53±2.04^B^	0.004±0.009
	TT(16)	343.77±159.15^A^^B^	1.99±6.41	-0.07±0.070^b^	8.94±4.48^B^	-0.035±0.025
	P value	0.0064*	0.7299	0.0401	0.0013**	0.4742
c.+2565 G>A	GG(477)	399.89±59.89^B^	4.08±2.70	-0.08±0.027^a^^b^	11.41±1.89^A^	-0.008±0.008
	GT(199)	204.99±67.21^A^	4.77±2.95	-0.02±0.030^a^	5.77±2.06^B^	0.004±0.009
	TT(12)	436.56±175.51^B^	3.57±7.05	-0.09±0.078^b^	8.62±4.92^A^^B^	-0.038±0.027
	P-value	0.0004**	0.9342	0.0138	0.0002**	0.4463

^1^ MY: milk yield, FY: fat yield, FP: fat percentage, PY: protein yield, PP: protein percentage.

^a,b^ within the same column with different superscripts means *P<* 0.05

^A,B^ within the same column with different superscripts means P*<* 0.01; The significant level after Bonferroni correction for multiple testing at P < 0.05 and P < 0.01 was 0.0071 and 0.0014, respectively.

*P indicates the significant association after Bonferroni correction for multiple testing at the significance level α = 0.05

**P indicates the significant association after Bonferroni correction for multiple testing at the significance level α = 0.01.

**Fig 2:**
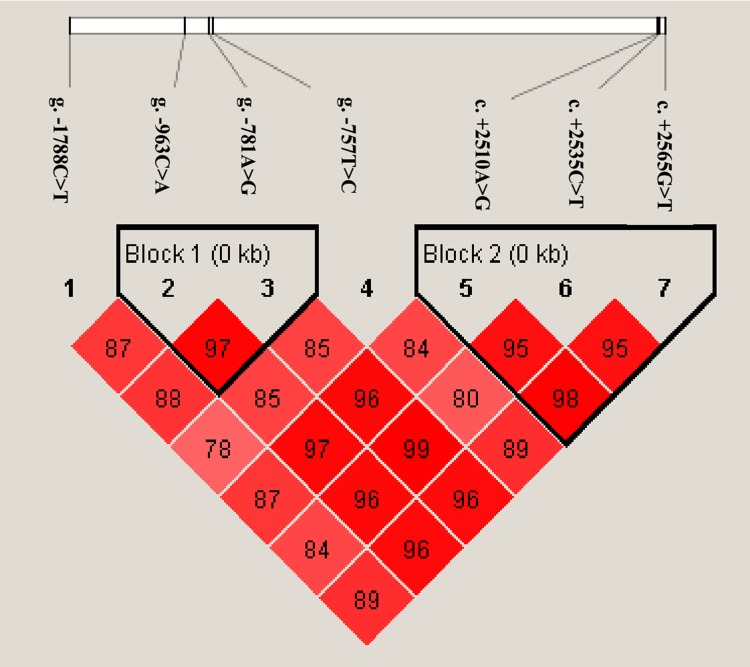
LD pattern for the genotyped SNPs within the *SAA1* gene. The darker shading indicates higher linkage disequilibrium and the number within rhombus was D' value.

With Haploview 4.2, two haplotype blocks were inferred as shown in Fig 2. The block1 was formed by g.-963C>A and g.-781A>G with D’ of 0.97. The block2 was composed of 3 SNPs, including c. +2510A>G, c. +2535C>T and c. +2565G>T, and the values of D’ among them were 0.95–0.98. Associations of the haplotypes with 5 milk production traits are presented in Tables A and B in S1 File.

### Regulation of the Promoter SNPs on Transcriptional Activity

**Fig 3:**
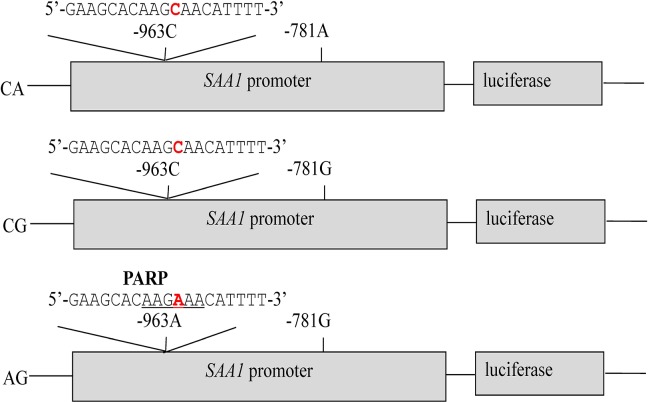
Transient reporter gene expression assays with constructs containing g.-963C>A and g.-781A>G in the *SAA1* gene and surrounding TFBS modified from TRANSFAC software output. Nucleotides underlined denote the TFBS sequences; the nucleotides in red highlight the position of the SNPs.

**Fig 4:**
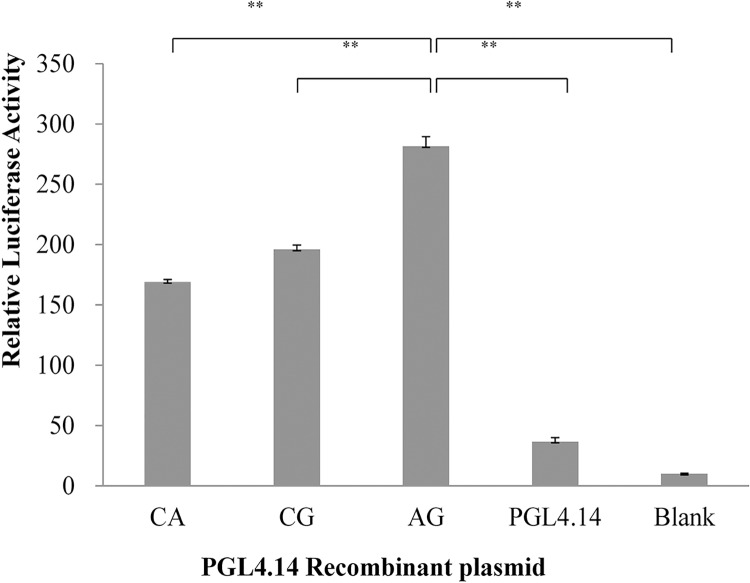
Luciferase activity analysis of the recombinant plasmids constructed by g. -963C>A and g. -781A>G in HEK293 cells. The value of each construct is the mean SEM for three independent experiments, each of which was performed in triplicate. *P* values are from a *t*-test (two-tailed). **P< 0.01.

As shown in Figs 3 and 4, the promoter activity of all three constructs was significantly higher than that of the pGL4.14 vector (*P* <0.01). The transcriptional level of haplotype AG was significantly higher than haplotypes CA and CG (*P* <0.01). These results suggested clearly indicated that the A allele in g.-963C>A up-regulated the transcriptional activity of the *SAA1* promoter, while g.-781A>G did not impact the transcription (*P* >0.05).

### Identification of TFBSs at the g.-963 C>A by EMSA

**Fig 5:**
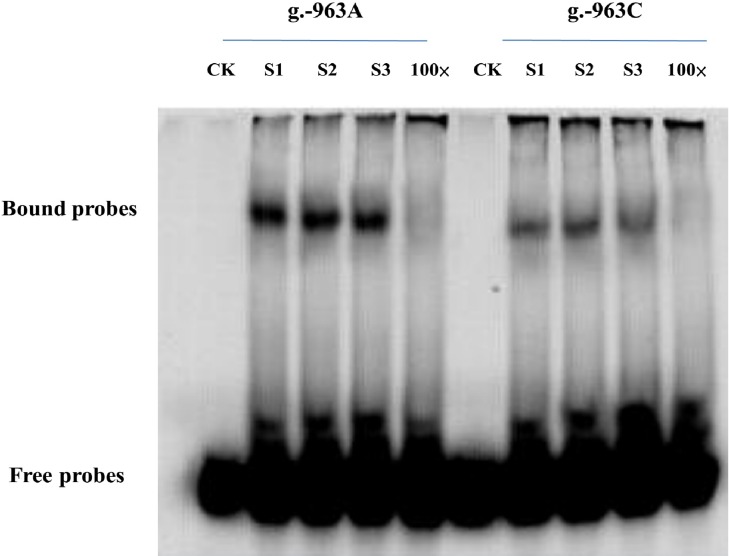
EMSA with wt and mutant oligonucleotides corresponding to the g. -963C>A site. CK, blank control; S1-3, sample 1–3; g. -963A, g. -963C showed that EMSA with wt and mutant oligonucleotides for the site g. -963C>A. 100× indicated a 100-fold excess of an unlabeled consensus oligonucleotide probe was used.

EMSA was performed to determine the above luciferase assay results. A pair of 5’-biotinylated oligonucleotide probes that contained either the wild-type sequences or the mutated bases were generated. As shown in Fig 5, the wild type A probe of g.-963 C>A visualized a DNA-protein complex while the mutated type C probe showed a weaker complex, confirming a binding of transcription factor in the allele A of g.-845 G>A. Whereas, the allele C and A of g.-963 C>A were shown to have undetectable DNA-protein binding (Fig 5).

### TFBSs Prediction

It was predicted that g.-963C>A created a TFBS, Poly ADP-ribose polymerase (PARP), while its substitution by A allele did not. The other 3 SNPs (g.-1788C>T, g.-781A>G and g.-757T>C) in the promoter did not change the binding of any TFBSs. Therefore, g.-963C>A polymorphism might affect the binding affinity of the surrounding sequences with the PARP and may be responsible for the activity of *SAA1* promoter.

### Non-Synonymous Functional Prediction

The alterations in the secondary structure of the *SAA1* mRNA caused by the non-synonymous mutation c. +2510A>G were predicted, which showed no difference in mRNA structure. Additionally, the effect of this mutation was predicted to be ‘tolerated’ by the SIFT programs, and the protein function might not be influenced. The results from the SOPMA server revealed that alpha helix were found to be changed predominantly from 51.54% to 53.08% by the substitution Gly/Asp (c. +2510A>G; G/A).

## Discussion

This study was a follow-up work to our previous RNA-seq work, in which the *SAA1* gene was detected to differentially express in the mammary gland between the high and low groups for milk protein and fat percentage of lactating Holstein cows [11]. By association analysis and function validation, we found that two SNPs, g.-963C>A changing the promoter activity and c. +2510A>G changing protein secondary structure, could be causative mutations. Our findings provided the first evidence for significant associations of the *SAA1* gene with milk production traits in dairy cattle.

Genetic variations in promoter regions may cause significantly potential phenotype diversity [22–23]. Coincided with these observations, we found that g.-963C>A and g.-781A>G loci in promoter region significantly associated with MY and PY. Individuals with genotypes CC and AA had higher MY or PY than those with genotypes AA and GG. Numerous studies have reported that the polymorphisms in the promoter region are associated with gene expression by altering putative transcription factor-binding sites [24–28]. In the present study, luciferase assay and EMSA analysis clearly demonstrated that the allele A of g.-963C>A in the promoter of the *SAA1* gene was associated with increased the promoter activity possibly initiated by binding the PARP factor while allele C did not. PARP, as a transcription factor, plays important roles in regulating gene expression involved in DNA damage repair, cell cycle and metabolism [29–32]. Furthermore, PARP has been identified as a co-activator of several transcription factors to participate in the activation of NF-κB [33].

Considering the significant association effects of g.-963C>A on milk yield and milk protein yield traits and its impact on transcription activity, it is possible that g.-963C>A might be the causative mutation of *SAA1*, which regulates the *SAA1* expression by changing the binding status of the transcription factor, PARP, to impact the formation of milk and protein traits. As for the loci g.-781A>G, its impact on transcription activity did not reach significance level. Hence, it was excluded to be a potential causative mutation. The significant association of g.-781A>G with MY and PY may be due to the very strong LD between g.-781A>G and g.-963C>A (D’ = 0.97). These findings suggest that the locus g.-963C>A is directly responsible for the *SAA1* expression and would be applied in marker-assisted selection.

On the other hand, the SNP c. +2510A>G was a missense mutation, leading to an amino acid substitution (p. Gly48Asp). Generally speaking, non-synonymous SNPs might be acting through influencing protein stability and/or function. Association study showed that this SNP was significantly associated with MY, FP and PY (0.00002 < P < 0.0004) Further prediction using the SOPMA server revealed that the alpha helix was changed from 51.54% to 53.08% by the substitution Gly/Asp (G/A). Because the alpha helix was preferably located at the core of the protein and had important functions in proteins for flexibility and conformational changes [34], it was presumed that the SAA protein was more stable in conformation when the base was replaced by G. Hence, the SNP c.+2510A>G might be another potential functional mutation.

Because synonymous SNPs (sSNPs) do not change the amino acids sequences, this type of genetic variations is not expected to change the structure and function of the related proteins. However, accumulating evidence indicates that sSNPs could be acting through influencing the conformation and stability of pre-mRNAs or protein expression [35–36]. Besides, sSNPs may influence gene expression by different codon usage [37–38]. In the present study, c.+2535C>T and c.+2565G>T were actually sSNPs. However, considering their significant effects on MY and PY, we inferred the most important reason was the very strong LD with the missense mutation c. +2510A>G (the values of D’ was 0.95 and 0.98, respectively). Also possibly, the two sSNPs (c. +2535C>T and c. +2565G>T) identified in *SAA1* also might be involved in the process of milk production through altering or increasing the mRNA stability.

In cattle, the *SAA1* encodes the acute phase protein serum amyloid A (*SAA*) which is mainly synthesized in the liver during inflammatory response [39]. However, Mahony et al (2006) also showed that SAA did not significantly influence milk amyloid A and the latter was correlated with with cell-based indicators of intramammary inflammation [40]. In addition, previous study reported that *SAA1* might be involved in the development of the mammary gland by an NF-κB-dependent signaling pathway [41]. Over-expression of *SAA1* suppressed growth of mammary epithelial cells [42]. In this study, integrated analysis of differential gene expression, phenotype data, and transcriptional activity indicated that *SAA1* may play a negative regulation role in the milk production traits in dairy cattle. Our results suggested that the *SAA1* may be an important gene for milk production traits in dairy cattle.

## Conclusion

The present study confirmed that the *SAA1* gene had significant association effects on milk yield and composition traits in dairy cattle. A total of 7 SNPs were identified in *SAA1* and 6 out of them were observed to be significantly associated with milk yield and composition traits. Further function analysis indicated that the loci g.-963C>A and c. +2510A>G could be the causal mutations contributed to the observed associations through altering promoter transcriptional activity and protein conformational changes. Our findings indicate that the identified variants are helpful for advanced marker-assisted selection in dairy cattle population. Further in-depth investigations are required to validate the biological mechanisms of the *SAA1* gene on the formation of milk production traits in dairy cattle.

## Supporting Information

S1 File This file contains associations of the haplotypes with 5 milk production traits. **Table A**, Associations of the haplotype combinations formed by g. -963C>A and g. -781A>G in block 1 with milk production traits in Chinese Holsteins. **Table B**, Associations of the haplotype combinations formed by c. +2510A>G, c. +2535C>T and c. +2565G>A in block 2 with milk production traits in Chinese Holsteins.
(DOCX)
