# Supplementary material for: Identification of Genetic Associations and Functional Polymorphisms of SAA1 Gene Affecting Milk Production Traits in Dairy Cattle
Source: PLoS One. 2016 Sep 9;11(9):e0162195. doi: 10.1371/journal.pone.0162195 (PMC5017741; doi:10.1371/journal.pone.0162195)
Supplement: S1 File — Table A, Associations of the haplotype combinations formed by g. -963C>A and g. -781A>G in block 1 with milk production traits in Chinese Holsteins. Table B, Associations of the haplotype combinations formed by c. +2510A>G, c. +2535C>T and c. +2565G>A in block 2 with milk production traits in Chinese Holsteins. (DOCX) [file pone.0162195.s001.docx]

**Table A.** Associations of the haplotype combinations formed by g. -963C>A and g. -781A>G in block 1 with milk production traits in Chinese Holsteins (LSM±SE).

| Haplotype combination  (No. individuals) | MY | FY | FP | PY | PP |
| --- | --- | --- | --- | --- | --- |
| H1H1 (269) | 520.98±64.87**^A^** | 9.17±2.88**^a^** | -0.08±0.030 | 16.35±2.02**^Aa^** | 0.007±0.009 |
| H1H2 (201) | 372.68±67.34**^B^** | 5.26±2.96**^ab^** | -0.07±0.031 | 11.96±2.07**^Ab^** | -0.003±0.010 |
| H1H3 (164) | 292.77±70.43**^B^** | 9.67±3.07**^a^** | -0.01±0.032 | 8.19±2.15**^BC^** | -0.006±0.010 |
| H2H3(66) | 55.59±103.30**^C^** | -1.64±4.27**^b^** | -0.03±0.046 | 3.23±2.99**^C^** | -0.007±0.016 |
| P value | **0.00003^**^** | 0.0106 | 0.0343 | **0.000****06^**^** | 0.8354 |

H1 = CA; H2 = CG; H3 = AG. Means in the same column with different lowercase superscripts are different at P<0.05; means in the same column with different uppercase superscripts are different at P<0.01. *P indicates the significant association after Bonferroni correction for multiple testing at the significance level α = 0.05; **P indicates the significant association after Bonferroni correction for multiple testing at the significance level α = 0.01.

**Table B.** Associations of the haplotype combinations formed by c. +2510A>G, c. +2535C>T and c. +2565G>A in block 2 with milk production traits in Chinese Holsteins (LSM±SE).

| Haplotype combination  (No. individuals) | MY | FY | FP | PY | PP |
| --- | --- | --- | --- | --- | --- |
| H1H1 (493) | 432.00±59.29**^A^** | 4.77±2.68 | -0.08±0.027**^A^** | 12.54±1.87**^A^** | -0.006±0.008 |
| H1H2 (207) | 232.13±67.16**^B^** | 5.02±2.95 | -0.02±0.031**^B^** | 6.46±2.06**^B^** | -0.004±0.010 |
| P value | **0.00002^**^** | 0.8973 | **0.0043^**^** | **0.00007^**^** | 0.7551 |

H1 = ACG; H2 = GTA. Means in the same column with different lowercase superscripts are different at P<0.05; means in the same column with different uppercase superscripts are different at P<0.01. *P indicates the significant association after Bonferroni correction for multiple testing at the significance level α = 0.05; **P indicates the significant association after Bonferroni correction for multiple testing at the significance level α = 0.01.
